# Effect of Harvest Time and Microbial Anaerobic Fermentation at Ruminal Degradability, In Vitro Digestibility to Milk Production and Milk Quality for Whole Plant Zhang Hybrid Millet in Dairy Cows

**DOI:** 10.3390/ani9100749

**Published:** 2019-09-29

**Authors:** Yujia Tian, Xuewei Zhang, Shengli Li, Kai Liu, Peng Guo

**Affiliations:** 1 Tianjin Key Laboratory of Agricultural Animal Breeding and Healthy Husbandry, College of Animal Science and Veterinary Medicine, Tianjin Agricultural University, Tianjin 300384, China; 15810814632@163.com (Y.T.); zhangxuewei63@163.com (X.Z.); 2 State Key Laboratory of Animal Nutrition, College of Animal Science and Technology, China Agricultural University, Beijing 100193, China; caulk@163.com; 3 Beijing Hao You Xun Tian Biotechnology Limited Liability Company, Beijing 100081, China; ailder@sina.com

**Keywords:** hybrid millet, whole Zhang hybrid millet, protein nutritive value, harvest time, microbial anaerobic fermentation

## Abstract

**Simple Summary:**

With its drought tolerant and high productive characteristics, Zhang hybrid millet is becoming an important food source for both humans and animals. Whole plant Zhang hybrid millet has great potential in lowering feed cost while providing relatively high crude protein, vitamin, and mineral content. This study aimed to disclose whether harvest time and microbial anaerobic fermentation can improve ruminal degradability and intestinal digestibility of whole Zhang hybrid millet, and explore the effect of microbial anaerobic fermented whole Zhang hybrid millet as feedstuff on milk yield and milk quality. Results showed that the interaction effect of harvest time and microbial anaerobic fermentation had significant influence on the crude ash, Acid detergent fiber (ADF), water-soluble carbohydrate (WSC), and neutral detergent insoluble protein (NDIP) content and effective degradability of dry matter (EDDM) of whole Zhang hybrid millet. Early harvested whole Zhang hybrid millet with microbial anaerobic fermentation appeared to have a better chemical profile with lower content of crude ash, Neutral detergent fiber (NDF), ADF, and higher content of WSC, and a better ruminal degradability with lower EDDM, effective degradability of neutral crude protein EDCP, and effective degradability of neutral detergent fiber (EDNDF). Microbial anaerobic fermented whole Zhang hybrid millet as feedstuff did not significantly affect milk components, but significantly reduced somatic cell count ( SCC) compared with controls. Milk yield was numerically higher in whole Zhang hybrid millet groups. Collectively, harvest time and microbial anaerobic fermentation could further improve the nutritive value of whole Zhang hybrid millet. Microbial anaerobic fermented whole Zhang hybrid millet as an alternative feedstock for dairy diet was safe and feasible.

**Abstract:**

This study assessed whether harvest time and microbial anaerobic fermentation could affect ruminal degradability and intestinal digestibility of whole Zhang hybrid millet, and estimate the effect of microbial anaerobic fermented whole Zhang hybrid millet as feedstuff on milk yield and milk quality. Protein degradation and intestinal digestion were determined using *in situ* nylon bag technique and three-step *in vitro* method, respectively. Results showed that harvest time, microbial anaerobic fermentation, or their interaction significantly affected EDDM, EDCP, and EDNDF (*p* < 0.05). In vitro fermentation was significantly influenced by harvest time. Early harvested samples appeared to have higher Total volatile fatty acid (TVFA) and lower acetate: propionate ratio than late harvested ones (*p* < 0.01). However, significant effect of harvest time and fermentation was failed to find in the estimation of rumen-undegradable protein (RUP) (*p* > 0.05). Microbial anaerobic fermented whole Zhang hybrid millet as feedstuff provided similar milk compositions compared with controls, and it significantly reduced SCC (*p* = 0.04). Milk yield was numerically higher in whole Zhang hybrid millet groups. In conclusion, harvest time and microbial anaerobic fermentation could further improve ruminal utilization of whole Zhang hybrid millet. Whole Zhang hybrid millet could be an alternative feedstock for dairy cows with acceptable safety profile and potential benefit in milk production.

## 1. Introduction

With the development of dairy industry in China, the number of dairy cows on hand is increasing, correspondingly, the demand for forage is also increasing. As feed cost accounts for 60–70% of the total cost of dairy cow breeding, it is urgent to find a feedstuff with low cost and high utilization. In China’s dairy cattle breeding industry, the main forages used are Chinese wild rye grass, alfalfa hay, and tall oat grass. However, high quality alfalfa hay and tall oat grass mainly rely on import, which increases feeding cost. In addition, Chinese wild rye grass mainly comes from natural grassland, which often mixes with other forages. Moreover, the nutrient content of Chinese wild rye is unstable, as it can be easily affected by the nutrient content of grassland and climate. Thus, the lack of high quality forage provided us with a constant impetus to develop new type of forage. 

Zhang Hybrid millet, a hybrid foxtail millet (*Setaria italica var. germanica* (Mill.) Schred.) developed in Hebei province of China, is a new type of feed with high nutrient values. It is suggested that the content of crude protein, vitamin, and mineral in Zhang Hybrid millet are higher than other millet straws [1]. Investigation also showed that Zhang Hybrid millet had similar growing pattern as common millet, but with a better dry matter accumulation [2]. After its first launch in the year 2000, nine species of Zhang hybrid millet have been developed within two decades. As a hybrid millet, it has expressed heterosis in terms of genes and yield. A study that identified 740 differentially expressed genes between hybrid millet and its parent lines found that most of these genes were upregulated in the hybrid [3]. Increased yield of Zhang hybrid millet has been reported every year, and it has already reached more than 400 kg yield per thousand hectares in 2011. In addition, Zhang hybrid millet is featured as drought-tolerant and highly productive, making it well adapted to arid regions. It has became an important food source for Chinese people, and nowadays, its use has been extended to dairy cow breeding. In previous research, Zhang hybrid millet as forage for dairy cattle showed little influence on milk components such as milk fat and milk protein, and an increasing trend in milk yield was observed, during which no adverse reactions have been observed in dairy cows [4,5].

The nutrient value of forage harvested in different period are quite different [6]. Harvest time is reported to alter the content of dry matter (DM) and neutral detergent fiber (NDF) in feedstuff [7,8]. When the quality of roughage is not ideal, it can be improved by fermentation, silage and alkalization. However, there are few reports on the nutritional quality differences of Zhang hybrid millet in different harvesting periods, and the need for microbial anaerobic fermentation of Zhang hybrid millet as a diet for dairy cows. 

Therefore, the purpose of this study was to compare whole Zhang hybrid millet at different harvest times, with or without microbial anaerobic fermentation in terms of (1) chemical profile, (2) *in situ* ruminal degradability, and (3) intestinal digestibility. The current study also aimed to discover the commercial value of Zhang hybrid millet in the feeding system of dairy cows by evaluating the effect of microbial anaerobic fermented whole Zhang hybrid millet in replacement of partial Chinese wild rye grass, alfalfa hay, or maize straw in the diet of dairy cattle on milk composition and milk yield. It was hypothesized that harvest time and microbial anaerobic fermentation would affect the chemical composition, ruminal degradability and intestinal digestibility of whole Zhang hybrid millet, and whole Zhang hybrid millet as feedstuff would affect milk components and production.

## 2. Materials and Methods

### 2.1. Feed Source and Processing

Five plots within one square metre block of land located in Hebei province of China were randomly chosen for collecting Zhang hybrid millet samples. Five plots were collected and served as repetitions. In 15 September 2016, 4 to 5 whole plant (stubble remaining: 6–8 cm) Zhang hybrid millets were randomly selected in each of the five spots. After harvesting, half of the samples were cut into 3–4 cm length, dried at 65 °C in a forced air oven for 48 h, and settled in room temperature for another 48 h to prepare hay. These samples were marked as early harvested hay (EH). The other half of the samples were cut into 5–8 cm length, dried at 65 °C to prepare hay for microbial anaerobic fermentation. The fermentation process was conducted according to the manufacture instruction (Beijing uni-haoyou Co., Ltd.: patent number: CN 201510289083.5, Beijing, China). Briefly, bacterial culture fluid (mainly contains *Lactobacillus uchneri*, *Aspergillus niger*, *Candida utilis*, and *Ligustrum reesei*) was activated in nutrient solution at 25–35 °C for 0.5–1.5 h. Bacterial culture fluid that contained up to 2 billion/mL of bacteria 2 h after activation was selected for fermentation. The samples were sprayed with bacterial culture fluid, kept in barrels (5 L each) to maintain anaerobic environment, cultured at 30 °C, 40–60% humidity for 7 days. After fermentation, samples were dried at 65 °C in a forced air oven for 48 h, and settled in room temperature for another 48 h. Early harvest hay was abbreviated as EH, early harvested sample with fermentation was marked as EF. The same amount of samples were collected at the same blocks after 30 days, the abovementioned processes were conducted and samples were marked as late harvested hay (LH) and late harvested hay with fermentation (LF), respectively. 

### 2.2. In Situ Rumen Incubation

Zhang hybrid millet samples for incubation test were prepared with the same method above to get EH, EF, LH, and LF. The final dry samples were ground to pass through a 2-mm sieve for in situ trial. All animal experiments in this study were approved by the Ethics Committee on Animals of Tianjin Agricultural University (TJAUA-2016-025). Four Holstein heifers of similar age and weight (20 months old, average body weight of 600 ± 30 kg) cannulated with a permanent rumen fistula with an internal diameter of 10 cm were used for measuring ruminal degradability of the four types of whole Zhang hybrid millet samples. A soft plastic cannula with a diameter of 0.5–0.8 cm and a length of 50 cm was fitted on the fistula. The heifers were individually fed twice daily at 06:00 and 16:00 to satisfy 1.3 times the maintenance nutrient requirement according to the National Research Council (NRC) nutrient requirement [9]. Water was available *ad libitum*. The diet formula and nutritional level were shown in Table 1. Before incubation, 7 g of samples were placed into numbered bags (monofilament open nylon wire) measuring 9 × 12 cm with pore size of 40 microns. A polyester mesh bag (45 × 45 cm with a 90 cm length of rope to anchor it to the cannula) with a plastic bottle (250 mL) filled with gravel was weighed down to keep the samples in the liquid strata of the rumen. Sample bags were added to the polyester mesh bag according to the “gradual addition/all out” schedule, and incubated for 72, 48, 24, 12, 8, 4 and 0 h. All sample bags for each incubation time were randomly allocated to the four heifers for two runs of incubation. After incubation, the bags were removed from the rumen, rinsed under cold tap water to remove excess ruminal contents and to stop microbial fermentation. The bags were washed with cool water without detergent and subsequently dried at 65 °C for 48 h. Dry samples were stored in a refrigerated room (4 °C) until chemical analysis.

### 2.3. In Vitro Rumen Fermentation

About 250 mL of rumen liquor was collected from three heifers two hours after feeding, respectively. The rumen fluid was strained through four layers of cheesecloth, evenly blended, and placed in an incubator. After mixing with buffer solution at a ratio of 2:1 (V/V), the rumen fluid mixture was incubated in a automated trace gas recording system (AGRS-1, Ruminant Nutrition Laboratory, China Agricultural University, Patent No. ZL200610011301.X) at 39 °C for 72 h. The in vitro buffer solution consisted of 400 mL distilled water, 200 mL mineral solution (5.7 g Na_2_HPO_4_ (Kermel, Guangzhou, China), 6.2 g KH_2_PO_4_ (Kermel, Guangzhou, China) and 0.6 g MgSO_4_·7H_2_O (Kermel, Guangzhou, China) mixture distilled with water to a constant volume of 1000 mL), 0.1 mL trace element solution (13.2 g CaCl_2_·2H_2_O (Kermel, Guangzhou, China), 10.0 g MnCl_2_·4H_2_O (Kermel, Guangzhou, China), 1.0 g CoCl_2_·6H_2_O (Kermel, Guangzhou, China) and 8.0 g FeCl_3_·6H_2_O (Kermel, Guangzhou, China) mixture distilled with water to a constant volume of 100 mL), 200 mL buffer solution (35 g NaHCO_3_ (Kermel, Guangzhou, China) and 4 g NH_4_HCO_3_ (Kermel, Guangzhou, China) mixture distilled with water to a constant volume of 1000 mL), and 40 mL reductant (4 mL 1 moL·L^−1^ NaOH (Kermel, Guangzhou, China) and 625 mg Na_2_S·9H_2_O (Kermel, Guangzhou, China) dissolved in 95 mL distilled water). Immediately after turning of the gas recording system, the pH of the incubation fluid was measured. Ammonia nitrogen (NH_3_-N) (Kermel, Guangzhou, China) was determined using a spectrophotometer as described by Broderick and Kang [10]. The incubation fluid and residual substrate were capped and placed into an auto sampler device (Agilent 7890A, Agilent Technologies Inc., Santa Clara, CA, USA) with a 30 m HP-INNOWax 19091N-213 (Agilent, CA, USA) capillary column (0.32 mm i.d. and 0.50 mm film thickness) for analysis. The rumen liquor was centrifuged at 4000 *g* for 15 min. The supernatant (20 mL) was collected and added with 3 mL of 25% metaphosphoric acid and 0.6% 2-Ethylbutyric acid. The volatile fatty acid (VAF) content of the rumen fluid was determined using gas chromatography described by Erwin et al. (1961) with some modification [11]. Using 2-ethyl butyric acid (Sigma-Aldrich, St. Louis, MO, USA) as internal standard, the assessment was carried out by gas chromatography (SP3420, Beijing Analytical Instrument Factory, Beijing, China) and glass-packed column. The chromatographic conditions are as follows: 6 mm × 2 m quartz glass-packed column (stationary phase 15% FFAP, Chromosorb 80–100 mesh), N-2000 chromatographic workstation; column temperature 150 °C, import temperature 200 °C, air pressure 200 KPa; injection volume 0.8 μL; FID detector temperature 220 °C; high purity N_2_ as carrier gas with a flow rate of 30 mL/min; H_2_ as gas with flow rate of 30 mL/min; air as auxiliary gas with a flow rate of 300 mL/min. 

### 2.4. Intestinal Digestion

Extra *in situ* rumen incubation was performed with the same method described above, only that samples were incubated for 16 h. Intestinal digestibility of rumen undegradable protein (RUP) was determined by using three-step *in-vitro* method described by Calsamiglia and Stern [12]. Briefly, residues at 16 h *in situ* rumen incubation time were collected, rinsed until the water was clear, dried at 55 °C for 48 h, and grounded to pass through a 1-mm screen Wiley mill. Then, 1 g of residues were weighted, placed into nylon bag, and incubated in HC1 solution (pH = 1.9) containing 1 g of pepsin (sigma, P-7000, Sigma Chemical, St. Louis, MO, USA) at 39 °C for 1 h. The samples from the five different blocks were set as repetitions, and all tests were run in triplicates. After incubation, the bags were rinsed under cool tap water until all the buffer were removed. Then, the bags were placed into culture bottle with 2 L of preheated trypsin solution (pH = 7.75, KH_2_PO_4_ buffer solution) (sigma P-7545, Sigma Chemical, St. Louis, MO, USA) containing 50 μg/L of thymol and 3 g of trypsin per liter, and shake cultured at 39 °C for 24 h. After that, the sample bags were rinsed again under tap water until the water was clear, and subsequently dried at 55 °C for 48 h before the measurement for N content.

### 2.5. Chemical Profile Analysis

All of the whole Zhang hybrid millet samples were analyzed for nutritional profiles based on the standard procedures described by Association of official analytical Chemists (AOAC) [13]. DM (AOAC method 930.15), crude ash (AOAC method 942.05), ether extract (EE, AOAC method 920.39), crude protein (CP, AOAC method 984.13) (8400 Kjeltec analyzer unit, Foss Tecator, city, country), acid detergent insoluble N (ADIN), and neutral detergent insoluble N (NDIN) were analyzed according to established procedures AOAC and the method Roe et al. (1990) suggested [14,15]. Water soluble carbohydrates (WSC) were determined according to the anthrone-sulfuric acid colorimetric method [16]. Calcium (Ca) concentrations were evaluated by the National Standard of the People’s Republic of China: Determination of Calcium in Feed (Chinese national standards/technique (GB/T) 6436-2002), and phosphorus (P) was by Determination of Phosphorus in Feed Spectrophotometry (GB/T 6437-2002). Gross energy (GE) was determined using a bomb calorimeter (model 6200; Parr Instrument Co., Moline, IL, USA). Organic matter (OM) was calculated as 100-ash. Acid detergent fiber (ADF), neutral detergent fiber (NDF), and acid detergent lignin (ADL) were analyzed by Ankom filter bag method (ANKOM 2000 Filter Bag technique, Ankom Technology, Fairport, NY, USA) [17]. NDF was analyzed without the addition of sodium sulfite and with the inclusion of heat stable α-amylase as previously described [17]. The amount of CP associated with NDF (NDIP) and ADF (ADIP) were determined by analyzing the Kjeldahl-N content of the NDF and ADF, respectively [18]. ADIP and NDIP were calculated as: ADIP = 6.25 × ADIN and NDIP = 6.25 × NDIN. The samples for amino acids (AA) analyses were hydrolyzed in constant with 6 NHCI at 110 °C for 24 h [19], evaporated under vacuum stream, the samples were then resuspended in the NA-S buffer and injected to the system. AA profiles were determined by HPLC technique, using a Beckman 7300 High Performance Amino Acids Analyzer (Beckman Coulter, Mannheim, Germany). AA were presented on g/16 g N, calculated as: amino acid content/(corresponding sample’s crude protein content/6.25).

### 2.6. Animal and Diet

Animal study was conducted at a dairy farm in Wuqiang County (Hengshui, Hebei province, China). Sixty dairy cows with milk production above 25 kg and days in milk (DIM) between 100 and 200 days were randomly selected and evenly divided into four groups. Basic characteristics of the selected samples, regarding milk production, month in milk and lactation number did not different significantly among the four groups. The cows were individually fed twice daily with total mixed rations (TMR) at 0800 and 1600 according to NRC requirement [9]. Feed was available *ad libitum*. The ration formula used in the four groups was based on the requirement of NRC with whole Zhang hybrid millet substituting different ratio of Chinese wild rye grass (50%), alfalfa hay (30%), or maize straw (20%). The detailed rations of each groups were summarized in Table 2. The microbial anaerobic fermented whole Zhang hybrid millets used in feeding trail were prepared with the same method described above. Briefly, after harvest, samples were cut into 5–8 cm length, dried at 65 °C to prepare hay for microbial anaerobic fermentation. Then, samples were fermented in barrels (5 L each) with bacterial culture fluid (Beijing uni-haoyou Co., Ltd.: patent number: CN 201510289083.5, Beijing, China) sprayed on the hay, cultured at 30 °C, 40–60% humidity for 7 days. After fermentation, the whole Zhang hybrid millets were directly used in TMR ration. Feed intake was measured as: (daily TMR - leftovers)/head count per group. The adaptation period to the diets was 15 days. After the adaptation period, dairy cows were fed for another month. Then, milk samples were collected continually for 3 days. Milk production was recorded twice a day at 0630 and 1800. Milk samples from each cow in each day was mixed at a ratio of 6:4 and kept in separate container. Each container contained about 40 mL milk. Before analysis, milk samples were added with potassium dichromate, and stored at a temperature of 4 °C. The content of milk fat, protein, lactose, non-fat dry solids, urea nitrogen, and somatic cell count (SCC) were determined within 1–2 days after collection. 4% fat corrected milk yield (kg/day) was calculated as: 0.4M + 15M × F. M is weight of milk in kg, and F is fat content of milk.

### 2.7. Calculation and Statistical Analysis

*Chemical profile and amino acids analysis.* Statistical analyses were performed using the MIXED procedure of SAS (version 9.2, SAS Inst. Inc., Cary, NC, USA) [20]. Yijk = µ + Si + Gj + SGij + ek (ij), where, Yijk is the observed value of the feed in the time and fermentation, µ is the mean value common to all observations, Si the fixed effects of the time, Gj the fixed effects of the fermentation, SGij the fixed interaction between the time and fermentation, and ek (ij) is the random deviation of the feed in the time and fermentation. The comparison of EH, LH, EF, and LF was carried out using the contrast statement in SAS. 

*Protein ruminal degradation.* Effective ruminal degradability were calculated based on the method suggested by method of Ørskov and McDonald (1979) [21,22] with the following formula: P = a + bc/(c+k), where a is the rapidly degraded fraction; b is potentially degraded fraction; c is fractional degradation rate of b; and the k value used in this study is uniformly 0.025 as suggested in Feng study [23]. Statistical analyses were performed using the MIXED procedure of SAS (version 9.2, SAS Inst. Inc., Carycity, NC, USA) [20]. The model used for the analysis was: Yijk = µ + Si + Gj + SGij + ek (ij), where, Yijk is the observed value of the feed in the time and fermentation, µ is the mean value common to all observations, Si the fixed effects of the time, Gj the fixed effects of the fermentation, SGij the fixed interaction between the time and fermentation, and ek (ij) is the random deviation of the feed in the time and fermentation.

*In vitro ruminal fermentation and intestinal digestible analysis*. Statistical analyses were performed using the MIXED procedure of SAS (version 9.2) [20]. The model used for the analysis was: Yijk = µ + Si + Gj + SGij + ek (ij), where, Yijk is the observed value of the feed in the time and fermentation, µ is the mean value common to all observations, Si the fixed effects of the time, Gj the fixed effects of the fermentation, SGij the fixed interaction between the time and fermentation, and ek (ij) is the random deviation of the feed in the time and fermentation.

*Milk quality and production test*. Effect of microbial anaerobic fermented whole Zhang hybrid millets on milk yield and milk composition were determined using the MIXED procedure of SAS (version 9.2) [20]. The model used for the analysis was: Yijk = µ + Ti + Sk +eijk, where, Yijk was an observation of the dependent variable ijk; µ was the population mean for the variable; Ti was the difference of the feed type, as a fixed effect, Sk was the run effect, as a random effect, and eijk was the random error associated with the observation ijk.

For all statistical analyses, significance was declared at *p* < 0.05 and a trends was considered at 0.05 < *p* ≤ 0.10. Differences among the treatments were evaluated using a multiple comparison test following the Tukey–Kramer method. Means with different letters were obtained with “pdmix800 SAS macro” [20].

## 3. Results 

### 3.1. Difference in Protein Chemical Profile among All Whole Zhang Hybrid Millet Samples

The detailed chemical profile were presented in Table 3. Harvest time and microbial anaerobic fermentation affected the chemical composition of whole Zhang hybrid millet. EH and EF samples contained significant lower crude ash, CP, NDF, and ADF, and higher EE and ADIP than LH and LF (*p* < 0.01), indicating that early harvest might result in more fibers and organic matter, but less protein content. EF and LF samples resulted in a lower DM (*p* < 0.01), and a higher water-soluble carbohydrate (WSC) (*p* < 0.05) when compared to EH and LH. Parameters concerning structural carbohydrate and crude protein profile were also differed between samples with or without microbial anaerobic fermentation. LF had higher ADF than LH (*p* < 0.05). EF and LF were higher in ADIP (*p* < 0.01) and ADL (*p* < 0.01), but lower in NDIP (*p* < 0.001) compared with EH and LH samples. The levels of crude ash (*p* < 0.05), ADF (*p* < 0.05), NDIP (*p* < 0.01) and WSC (*p* < 0.05) showed strong interactions between harvest time and microbial anaerobic fermentation. EF group contained a lower levels of crude ash, CP, ADF and NDIP, and a highest value of WSC than EH, LH, and LF groups.

### 3.2. Difference in Amino Acid among All Whole Zhang Hybrid Millet Samples

Table 4 summarizes the results of amino acids (AA) in whole Zhang hybrid millet samples. EH and EF groups were significantly lower in proline (*p* < 0.05), and higher in glutamic (*p* < 0.01), alanine (*p* < 0.01), and leucine (*p* < 0.01) than that of the LH and LF samples. EF and LF had lower content of aspartic acid *(p < 0.05)* than EH and LH. Harvest time appeared to have significant influence on the majority of the assessed AA, except tyrosine, histidine, and arginine (*p* > 0.05). Microbial anaerobic fermentation treatment also seemed to affect the content of AA in whole Zhang hybrid millet samples. However, the interaction effect between harvest time and microbial anaerobic fermentation failed to manifest in almost all of the AA profiles, except for aspartic acid and arginine (*p* = 0.02). EF group appeared to have the highest glutamic, alanine, and leucine content among the four groups.

### 3.3. Difference in In Situ Ruminal Degradation

The effects of harvest time and microbial anaerobic fermentation on *in situ* rumen effective degradability (ED-) of DM (EDDM), CP (EDCP), OM (EDOM), and NDF (EDNDF) were presented in Table 5. Results showed that harvest time and microbial anaerobic fermentation did not influence the EDDM and EDOM in the rumen. However, EDCP and EDNDF had strong correlation with harvest time. EH and EF were lower in EDCP (*p* < 0.05) and EDNDF (*p* < 0.05) compared with LH and LF. Microbial anaerobic fermentation only affected the EDCP. EF and LF appeared to have lower EDCP than EH and LH (*p* < 0.05). The interaction of harvest time and microbial anaerobic fermentation had no significant effect (*p* > 0.05) on the in situ rumen effective degradability. EDDM (35.05%), EDCP (31.87%), and EDNDF (23.45%) of EF group appeared to be the lowest among the four groups.

### 3.4. Difference in In Vitro Ruminal Fermentation and Intestinal Digestion

Results from Table 6 demonstrated that both harvest time and microbial anaerobic fermentation had no significant effect on ammoniacal nitrogen in in vitro ruminal fermentation (*p* > 0.05). pH was significantly affected by harvest time and microbial anaerobic fermentation (*p* < 0.05), LF appeared to have a highest pH values among the four groups. Although pH was significantly affected (*p* < 0.05) by harvest time and microbial anaerobic fermentation, the value in each group was within the normal range (5.9–6.8). Harvest time affected nearly all measured parameters except valerate. The values of acetate, propionate, isobutyrate, butyrate, and isovalerate in EH and EF groups were higher than that of LH and LF (*p* < 0.01). EH and EF samples also resulted in a better total volatile fatty acids (TVFA) (*p* < 0.01), and a lower acetate: propionate ratio (*p* < 0.01) compared with LH and LH. Microbial anaerobic fermentation treatment seemed to have little effect on in vitro ruminal fermentation. The interaction effect of harvest time and microbial anaerobic fermentation did not exert in volatile fatty acids (VFA) and acetate: propionate ratio (*p* > 0.05). The intestinal digestibility of rumen undegradable protein (RUP) was not affected (*p* > 0.05) by harvest time, microbial anaerobic fermentation, and their interaction effect.

### 3.5. Difference in Milk Quality and Production

The effect of microbial anaerobic fermented whole Zhang hybrid millet substituting 50% Chinese wild rye grass, 30% alfalfa hay or 20% maize straw of normal feedstock on milk quality and milk yield were presented in Table 7. Milk protein in control and the three substitution groups were 3.53%, 3.50%, 3.66%, 3.53%, respectively, and the content of urea nitrogen ranged from 10.88 to 11.88 mg/100 mL. No significant difference was observed among groups regarding milk yield, milk fat, protein, lactose, non-fat dry solids, and urea nitrogen (*p* > 0.05). Yet, milk production in Chinese wild rye grass (22.24 kg/d), alfalfa hay (23.09 kg/d), and maize (22.43 kg/d) substitution groups were numerically higher than controls (21.40 kg/d). SCC in all three substitution groups (Chinese wild rye grass: 176.61 × 10^3^ cells/mL; alfalfa hay: 176.69 × 10^3^ cells/mL; maize: 110.51 × 10^3^ cells/mL) was significantly lower than that of the control group (245.66 × 10^3^ cells/mL). 

## 4. Discussion

As a novel forage, Zhang hybrid millet is suggested to have good feeding effect for cow and sheep when replacing Chinese wild rye grass or maize straw in the TMR diet [24,25]. Yet, the nutrient value of Zhang hybrid millet can be further improved by the management of harvest time or the use of microbial anaerobic fermentation. Previous studies demonstrated that roughages harvested at different time within a day, a month, or a season could affect the content of DM, WSC, CP, and NDF [7,8,26,27]. Results from the present study indicated that early harvest (15 September) resulted in more organic matter, and less fibers in Zhang hybrid millet. Microbial anaerobic fermentation could increased the content of WSC. The possible reason for this phenomenon was that the fermentation process could destroy the plant cell wall under the action of bacteria, and then expose the nutrients in plant stalks, resulting in the increase of WSC content at the evaluation. On the other hand, this phenomenon also suggested that microbial anaerobic fermentation was beneficial to increase the available carbohydrate content of Zhang hybrid millet. Our finding seemed to contrast with what was generally known about fermentation, which was bacteria would reduce WSC content in feed. However, the bacterial culture fluid used in this study was a specific patent developed for Zhang hybrid millet. The results of this study might be because although there are many complex bacteria in the solution, not all of these are nutrient-consuming bacteria. In the fermentation process, microorganisms might cause unpredictable impact on feedstuff, or there were special mechanism between this type of bacterial culture fluid and Zhang hybrid millet, which is worth further investigation. Collectively, early harvest Zhang hybrid millet with microbial anaerobic fermentation (EF) might provide a better feeding value for dairy cow with higher carbohydrate and lower fiber. Furthermore, the energy (16.71MJ/kg), CP (8.93%), NDF (52.77%), ADF (28.59%) and crude ash (6.91%) content of EF samples were superior to Chinese wild rye grass and straw forage, and similar to high quality oat grass. Taking nutrient components as evaluation index, whole plant Zhang hybrid millet could be used as high-quality gramineous forage in the diet of dairy cows.

On a practical basis, dietary CP is often overfed to ensure a sufficient supply of all AA to support all biological functions. Lowering CP intake by adequate balancing for AA could overcome over N or NH3 pollution, decrease pollution of water and soil, reduce feed cost, and improve herd performance and reproduction of dairy cow [28,29]. This can be achieved by the selection of harvest time and the application of microbial anaerobic fermentation. Both factors and their interaction effect had significant influence on many of the AA content. In general, EF group might be a better protein source for dairy cow, as it contained a relatively low CP profile, however, some of the AA content were relatively high among the four groups of sample. Since AA would be degraded in the rumen, it is speculated whether the difference in dietary AA content has positive effect on milk production. However, a meta-analysis evaluating two subtly different hypotheses using different statistical approaches had found good consistency in the AA identified as being associated with production [30]. It was suggested that leucine, histidine, and threonine was positively correlated with milk protein and milk yield. Thus, these AA should be given greater consideration in diets. This further approved that early harvest time and microbial anaerobic fermentation could be a useful way to provide high nutrient Zhang hybrid millet that eventually improved milk production. 

The rumen effective degradability of Zhang hybrid millet was affected by harvest time and microbial anaerobic fermentation. Early harvested Zhang hybrid millet with microbial anaerobic fermentation could appropriately reduce the degradation rate (DM, OM, NDF) in rumen, on the other hand, increase the ratio of rumen by-pass protein to the intestine, which provide better conditions for intestinal digestion [31]. In addition, TVFA concentration in in vitro rumen fluid was higher for EF despite lower in situ EDDM, EDOM and EDNDF. It was possible that although the rumen effective degradation rate (in situ experiment) was low, the degraded parts could be widely used by microorganisms in the rumen thereby metabolize volatile acid. 

Previous analysis showed better chemical composition, in situ ruminal degradability, and in vitro fermentation in microbial anaerobic fermented Zhang hybrid millet. Thus, a feeding trial was conducted to explore the feasibility of microbial anaerobic fermented Zhang hybrid millet as feedstuff for dairy cow. There would be some odor emitting after fermentation. In order to avoid affecting the palatability of dairy cow, microbial anaerobic fermented Zhang hybrid millet were designed to replace 50% of Chinese wild rye grass, 30% of alfalfa hay, or 20% of maize straw in TMR. In addition, the replacement ratio was calculated according to the nutritional requirements, and aimed to balance the energy and protein content among the four groups of feed. Results of milk composition suggested that the substitution groups could produce milk with sufficient energy and protein as the control group, and the milk products were safe and reliable. Also, the use of microbial anaerobic fermented Zhang hybrid millet did not reduce milk yield, suggesting that it was acceptable in dairy cow feeding. Furthermore, SCC in Zhang hybrid millet substitution groups was significantly lower than controls. By far, there are nearly none of the publication suggesting the influence of Zhang hybrid millet on immune function of dairy cow. We assume that this type of fermented Zhang hybrid millet might acquire some distinct compounds that have some effect on improving the health and immunity of dairy cows. However, the immune indexes of dairy cows were not determined in this experiment, which could be further improved in future related studies. In addition, the lower SCC might be a result of the low NDF content in microbial anaerobic fermented Zhang hybrid millet [32]. As lower SCC was linked to higher milk yield in a number of studies [33,34], the use of microbial anaerobic fermented Zhang hybrid millet might also possess potential in increasing milk production. However, this study failed to confirm this connection.

Altogether, the current study was the first attempt to assess the feasibility of microbial anaerobic fermented Zhang hybrid millet in diary cow feeding. Future studies applying larger proportion of microbial anaerobic fermented Zhang hybrid millet in the diet of dairy cow are needed to verify its effect on milk yield and quality. Additionally, in the process of microbial anaerobic fermentation, it was found that if the grains shed too much, it might affect the nutritional components. Thus, in future experiments, attention should be paid to reducing grain shedding during microbial anaerobic fermentation to avoid affecting the fermentation effect.

## 5. Conclusions

It was concluded that harvest time and microbial anaerobic fermentation treatment could affect the chemical profile, *in situ* ruminal degradation, and in vitro fermentation of whole plant Zhang hybrid millet. Early harvested whole Zhang hybrid millet with microbial anaerobic fermentation treatment obtained a better nutrient components and ruminal fermentation effect. The use of the two approaches could help achieve the maximum use of whole plant Zhang hybrid millet in dairy cow feeding. The use of microbial anaerobic fermented whole Zhang hybrid millet in substitution of Chinese wild rye grass, alfalfa hay, or maize straw in dairy diet provided high-quality milk product and similar milk yield as common TMR, suggesting that whole Zhang hybrid millet could be an alternative feedstock for dairy cows. Zhang hybrid millet features as high yield and low in price. The use of Zhang hybrid millet as feedstuff can potentially reduce feed cost and eventually increase economic benefits. Nevertheless, the present study was the first attempt to assess the feasibility of microbial anaerobic fermented Zhang hybrid millet in diary cow feeding. Further studies are required to verify the actual effect of Zhang hybrid millet. 

## Figures and Tables

**Table 1:** Ingredients and nutritional level of diet.

Ingredient (DM basis)	% DM	Nutritional Level	Content
Alfalfa hay	45.0	NE_L_ * (MJ/kg)	5.63
Maize	22.1	CP (%)	15.9
Chinese wild rye grass	15.0	Ca (%)	0.72
Cottonseed meal	6.38	P (%)	0.43
Soybean meal	5.51		
Rice bran	4.76		
Salt	0.42		
Sodium hydrogen phosphate	0.42		
Additive premix	0.42		

CP, crude protein. DM, Dry matter. * The nutrient levels in the table were actual measured value except NE_L_.

**Table 2:** Ingredients and chemical composition of dietary treatments (DM basis).

Ingredient (kg)	Control	Substituted 50% of Chinese Wild Rye Grass	Substituted 30% of Alfalfa Hay	Substituted 20% of Maize Straw
Corn silage	22.0	22.0	22.0	22.0
Maize straw	4.50	4.50	4.50	3.60
Alfalfa hay	2.50	2.50	1.76	2.50
Soybean meal	2.20	2.20	3.00	2.20
Chinese wild rye grass	1.50	0.75	1.50	1.50
Extruded soybean	1.50	1.50	0.70	1.50
Others (Premix, mineral, etc.)	0.61	0.61	0.61	0.61
Whole Zhang hybrid millet	0.00	0.72	0.72	0.84
Dry matter intake	18.6	18.6	18.6	18.6
Chemical composition (% DM)				
Crude protein	15.6	15.7	15.5	15.6
Neutral detergent fibre	37.9	37.9	38.2	39.7
Acid detergent fibre	24.0	23.6	23.3	25.1
Calcium	1.06	1.06	1.06	1.10
Phosphorus	0.54	0.53	0.54	0.54
Net energy for lactation (MJ/kg)	6.39	6.48	6.41	6.30

DM, Dry matter.

**Table 3:** Effects of harvest time and microbial anaerobic fermentation treatment on chemical profile of whole Zhang hybrid millet.

Items	EH	EF	LH	LF	SEM	Contrast *p* Value		
						Harvest Time	Fermentation	Time × Fermentation
Basic Chemical Profile								
DM, g/kg	933.9 ^a^	925.5 ^b^	934.2 ^a^	926.4 ^b^	1.81	0.78	<0.01	0.89
Crude ash, g/kg DM	72.6 ^c^	69.1 ^d^	89.7 ^b^	98.9 ^a^	2.12	<0.01	0.26	0.02
EE, g/kg DM	22.6 ^a^	23.9 ^a^	12.8 ^b^	14.1 ^b^	0.72	<0.01	0.04	0.99
Structural Carbohydrate Profile								
NDF, g/kg DM	524.5 ^c^	527.7 ^c^	614.0 ^b^	647.1 ^a^	9.73	<0.01	0.13	0.21
ADF, g/kg DM	282.3 ^c^	285.9 ^c^	320.8 ^b^	364.6 ^a^	6.56	<0.01	0.01	0.02
ADL, g/kg DM	29.2 ^b^	41.9 ^a^	26.3 ^b^	43.1 ^a^	4.08	0.80	<0.01	0.57
Protein profile								
CP, g/kg DM	93.1 ^c^	89.3 ^d^	113.8 ^a^	105.3 ^b^	2.29	<0.01	0.03	0.39
NDIP, g/kg DM	15.4 ^b^	15.4 ^b^	21.6 ^a^	14.9 ^b^	0.73	<0.01	<0.001	<0.001
ADIP, g/kg DM	10.7 ^b^	13.4 ^a^	5.3 ^d^	7.9 ^c^	0.67	<0.01	<0.01	0.98
Gross Energy (MJ/kg)	16.14 ^ab^	16.71 ^a^	15.45 ^b^	16.10 ^ab^	0.334	0.04	0.05	0.90
WSC	3.08 ^c^	9.83 ^a^	8.45 ^b^	9.35 ^a^	1.108	0.04	0.02	0.02

EH, early harvest hay; EF, early harvest hay with fermentation; LH, late harvest hay; LF, late harvest hay with fermentation; DM, dry matter; EE, ether extract; NDF, neutral detergent fiber; ADF, acid detergent fiber; ADL, acid detergent lignin; cp, crude protein; NDIP, neutral detergent insoluble crude protein; ADIP, acid detergent insoluble crude protein; WSC, water-soluble carbohydrate. ^a–d^ Values in the same line with different capital letter superscripts mean samples have significant difference. The same as below. SEM = standard error of mean, the same as below.

**Table 4:** Effects of harvest time and microbial anaerobic fermentation treatment on amino acid content of whole Zhang hybrid millet.

Items (g/kg DM)	EH	EF	LH	LF	SEM	Contrast *p* Value		
						Harvest Time	Fermentation	Time × Fermentation
Aspartic acid	0.50 ^b^	0.43 ^c^	0.83 ^a^	0.43 ^c^	0.04	0.02	<0.01	0.02
Threonine	0.19	0.23	0.19	0.19	0.01	<0.01	0.03	0.19
Serine	0.23	0.25	0.21	0.18	0.01	<0.01	0.43	0.10
Glutamic	0.75 ^a^	0.88 ^a^	0.50 ^b^	0.56 ^b^	0.02	<0.01	0.01	0.34
Proline	0.34 ^b^	0.35 ^b^	0.60 ^a^	0.60 ^a,b^	0.04	0.02	0.05	0.06
Glycine	0.17	0.20	0.20	0.21	0.01	0.20	<0.01	0.96
Alanine	0.36 ^a,b^	0.45 ^a^	0.28 ^b,c^	0.34 ^c^	0.01	<0.01	<0.01	0.24
Valine	0.23	0.27	0.22	0.23	0.01	<0.01	<0.01	0.42
Isoleucine	0.19	0.23	0.17	0.17	0.01	<0.01	0.04	0.41
Leucine	0.51 ^a,b^	0.62 ^a^	0.36 ^c^	0.38 ^c^	0.02	<0.01	<0.01	0.09
Tyrosine	0.20	0.20	0.20	0.18	0.02	0.14	0.83	0.71
Phenylalanine	0.11	0.13	0.09	0.09	0.01	<0.01	0.40	0.28
Histidine	0.09	0.10	0.08	0.10	0.01	0.53	0.01	0.37
Lysine	0.17	0.19	0.21	0.23	0.01	<0.01	0.02	0.48
Arginine	0.15	0.19	0.19	0.17	0.01	0.66	0.34	0.02

EH, early harvest hay; EF, early harvest hay with fermentation; LH, late harvest hay; LF, late harvest hay with fermentation; DM, dry matter; ^a–c^ Values in the same line with different capital letter superscripts mean samples have significant difference. SEM = standard error of mean.

**Table 5:** Effects of harvest time and microbial anaerobic fermentation treatment on In situ rumen degradation of whole Zhang hybrid millet.

Items	EH	EF	LH	LF	SEM	Contrast *p* Value		
						Harvest Time	Fermentation	Time × Fermentation
In situ rumen degradation of DM								
a (%) ^1^	0.09 ^b^	0.49 ^b^	3.41 ^a^	4.12 ^a^	1.02	<0.01	0.54	0.86
b (%) ^2^	60.95 ^a^	56.14 ^a,b^	48.20 ^c^	52.06 ^b,c^	2.90	<0.01	0.85	0.10
c (h^−1^) ^3^	0.04 ^b^	0.04 ^b^	0.06 ^a^	0.04 ^b^	0.01	0.16	0.04	0.06
EDDM (%)	39.05 ^a^	35.05 ^b^	37.70 ^a,b^	37.76 ^a,b^	1.18	0.52	0.07	0.05
In situ rumen degradation of CP								
a (%)	3.16 ^a,b^	0.70 ^b^	10.00 ^a^	8.97 ^a^	2.72	<0.01	0.47	0.76
b (%)	55.84 ^a,b^	65.87 ^a^	42.06 ^a,b^	38.72 ^b^	9.85	0.03	0.70	0.44
c (h^−1^)	0.06 ^b^	0.03 ^b^	0.11 ^a^	0.06 ^b^	0.01	<0.01	<0.01	0.36
EDCP (%)	38.68 ^b^	31.87 ^c^	45.22 ^a^	38.18 ^b,c^	2.50	0.01	0.01	0.96
In situ rumen degradation of OM								
a (%)	0.03 ^b^	0.43 ^b^	2.98 ^a^	1.33 ^ab^	0.76	0.01	0.35	0.13
b (%)	57.59 ^a^	55.77 ^a^	46.12	47.47 ^b^	3.09	<0.01	0.93	0.56
c (h^−1^)	0.06	0.04	0.06	0.05	0.09	0.31	0.12	0.64
EDOM (%)	38.82	35.95	37.36	35.36	1.36	0.40	0.06	0.72
In situ rumen degradation of NDF								
a (%)	2.47 ^a,b^	6.42 ^a^	−0.73 ^b,c^	−2.16 ^c^	1.41	<0.01	0.41	0.09
b (%)	45.82	46.52	48.65	51.85	3.02	0.56	0.78	0.86
c (h^−1^)	0.04	0.02	0.04	0.04	0.13	0.52	0.38	0.30
EDNDF (%)	25.20 ^a,b^	23.45 ^b^	30.68 ^a^	31.12 ^a^	1.72	0.01	0.77	0.63

EH, early harvest hay; EF, early harvest hay with fermentation; LH, late harvest hay; LF, late harvest hay with fermentation; DM, dry matter; CP, crude protein; OM, organic matter; NDF, neutral detergent fiber; EDDM, effective degradability of dry matter; EDCP: effective degradability of crude protein; EDOM, effective degradability of organic matter; EDNDF, effective degradability of neutral detergent fiber. ^1–3^: a: rapidly degraded fraction; b: potentially degraded fraction; c: fractional degradation rate of b. ^a–c^ Values in the same line with different capital letter superscripts mean samples have significant difference. The same as below. SEM = standard error of mean, the same as below.

**Table 6:** Effects of harvest time and microbial anaerobic fermentation treatment on situ rumen fermentation and intestinal digestion of whole Zhang hybrid millet.

Items	EH	EF	LH	LF	SEM	Contrast *p* Value		
						Harvest Time	Fermentation	Time × Fermentation
pH	6.63 ^b^	6.65 ^b^	6.64 ^b^	6.71 ^a^	0.02	0.04	0.03	0.17
Ammoniacal nitrogen (mg/100 mL)	17.10	17.09	17.29	13.27	1.32	0.13	0.10	0.10
Acetate (mmol/L)	69.27 ^a^	69.08 ^a^	64.68 ^a,b^	58.96 ^b^	2.50	<0.01	0.19	0.22
Propionate (mmol/L)	25.76 ^a^	26.29 ^a^	22.40 ^b^	20.08 ^b^	0.86	<0.01	0.25	0.07
Isobutyrate (mmol/L)	1.76 ^a^	1.75 ^a^	1.59 ^b^	1.44 ^b^	0.06	<0.01	0.16	0.21
Butyrate (mmol/L)	12.95 ^a^	13.40 ^a^	11.23 ^b^	10.18 ^b^	0.47	<0.01	0.48	0.08
Isovalerate (mmol/L)	3.65 ^a^	3.74 ^a^	3.08 ^b^	2.86 ^b^	0.13	<0.01	0.59	0.20
Valerate (mmol/L)	1.78 ^a,b^	1.68 ^a,b^	2.01 ^a^	1.50 ^b^	0.14	0.86	0.02	0.10
TVFA (mmol/L)	115.27 ^a^	115.95 ^a^	104.98 ^b^	95.03 ^b^	3.92	<0.01	0.20	0.13
Acetate/propionate ratio	2.69 ^b^	2.63 ^b^	2.89 ^a^	2.94 ^a^	0.05	<0.01	0.91	0.27
Intestinal digestibility of RUP								
dRUP, %	36.15	28.96	33.40	32.94	2.07	0.85	0.25	0.31

EH, early harvest hay; EF, early harvest hay with fermentation; LH, late harvest hay; LF, late harvest hay with fermentation; TVFA, total volatile fatty acids; RUP, rumen undegradable protein; dRUP, intestinal digestibility of RUP. ^a,b^ Values in the same line with different capital letter superscripts mean samples have significant difference. SEM = standard error of mean.

**Table 7:** Effects of microbial anaerobic fermented whole Zhang hybrid millet on milk components and milk production.

Items	Control	Substituted 50% of Chinese Wild Rye Grass	Substituted 30% of Alfalfa Hay	Substituted 20% of Maize Straw	SEM	*p*-Value
Milk yield (kg/d)	21.40	22.24	23.09	22.43	0.91	0.52
4% fat corrected milk yield (kg/d)	21.35	20.59	22.64	22.71	0.93	0.12
Milk fat (%)	4.01	4.32	4.08	3.95	0.25	0.65
Protein (%)	3.53	3.50	3.66	3.53	0.16	0.85
Lactose (%)	4.96	4.92	4.94	4.89	0.04	0.59
Non-fat dry solids (%)	13.58	13.75	13.78	13.36	0.26	0.56
Urea nitrogen (mg/100 mL)	11.88	10.88	11.78	11.87	0.63	0.51
SCC (×10^3^/mL)	246 ^a^	174 ^ab^	177 ^ab^	111 ^b^	34.31	0.04

SCC, somatic cell count. ^a,b^ Values in the same line with different capital letter superscripts mean samples have significant difference. SEM = standard error of mean.
